# Innovation in Tigernut (*Cyperus Esculentus* L.) Milk Production: In Situ Hydrolysis of Starch

**DOI:** 10.3390/polym12061404

**Published:** 2020-06-23

**Authors:** Djomdi, Bakari Hamadou, Olivier Gibert, Thierry Tran, Cedric Delattre, Guillaume Pierre, Philippe Michaud, Richard Ejoh, Robert Ndjouenkeu

**Affiliations:** 1 Department of the Renewable Energies, The National Advanced School of Engineering of Maroua, University of Maroua, Maroua P.O.Box 46, Cameroon; hamadou.bakari@yahoo.fr; 2 Energy Research Laboratory, Renewable Energy Section (LRE/SENC), Institute for Geological and Mining Research (IRGM), Nlongkak Yaounde P.O.Box 4110, Cameroon; 3 UMR Qualisud, University of Montpellier, CIRAD, Montpellier SupAgro, University of Avignon, University of La Réunion, 73 rue JF Breton, 34398 Montpellier, France; olivier.gibert@cirad.fr (O.G.); thierry.tran@cirad.fr (T.T.); 4 The Alliance of Bioversity International and the International Center for Tropical Agriculture (CIAT), CGIAR Research Program on Roots Tubers and Bananas (RTB), Apartado Aéreo 6713, Cali 763537, Colombia; 5 Université Clermont Auvergne, CNRS, SIGMA Clermont, Institut Pascal, F-63000 Clermont-Ferrand, France; cedric.delattre@uca.fr (C.D.); guillaume.pierre@uca.fr (G.P.); 6 Depantment of Food Science and Nutrition, University of Ngaoundéré, Ngaoundéré P.O.Box 455, Cameroon; rabejoh@yahoo.com (R.E.); rndjouenkeu@yahoo.fr (R.N.)

**Keywords:** *Cyperus esculentus*, sprouting, carbohydrates, amylases, depolymerization, sweetness

## Abstract

Tigernut tubers (*Cyperus esculentus*) are used for the production of vegetable milk, commonly known as “Horchata de chufa” in Spain. The presence of starch in the tuber limits the yield of the milk, since this carbohydrate gelatinizes during the pasteurization of the milk and leads to the considerable solidification of this drink. The present work aims to improve the yields and extraction practice of the milk by an in situ hydrolysis of starch, using exogenous amylases of industrial or vegetable origin. The obtained results show that sprouting improves the extraction yields of tigernut milk, which goes from 50% to about 70%. This improvement in milk yield corresponds to a hydrolysis of about 35% of the starch in the tuber. The use of exogenous amylases leads to starch hydrolysis rates of 45% and 70%, respectively, for amylolytic extracts from sprouted tigernut tubers and amylase, with the corollary of a natural increase in the sweetness of milk. This technical approach makes it possible to produce a naturally sweetened tigernut milk which easily lends itself to pasteurization without a significant increase in viscosity.

## 1. Introduction

Tubers are foodstuffs that contain more starch, by dry weight, than almost all other food crops [1]. Many authors have shown that *Cyperus esculentus* L. is an edible plant which produces tubers containing an important starch quantity (about 30–40%) [2,3,4]. These tigernut tubers are used in Spain for the production of a milky drink called hochata de chufa [5,6]. This drink does not lend itself well to thermal sanitation treatments due to the gelatinization and retrogradation of its starchy component during pasteurization and cooling. This constraint induces, in the technical practices for producing horchata, either an elimination of the pasteurization operation of the milky drink, which reduces its capacity for preservation, or a complementary decanting operation to eliminate the starchy compound, with consequently reduces the nutritional value of the drink [7,8]. In both cases, sugar is added at the drink to improve the taste.

Various studies [3,7,9] have shown interest in the sprouting of tigernut tubers, especially in the production of malted flour. This operation leads to a hydrolysis of starch into free sugars, which can be used to reduce the risk of gelatinization of carbohydrate compounds during the production and pasteurization of the milky drink. In the same order of ideas, the in situ hydrolysis of the tigernut starch tubers can be considered by adding industrial amylases during the production of the beverage.

The objective of this study is therefore to improve the yield and extraction practice of tigernut milk; which constitutes an innovative opportunity through the production of this drink from sprouting tubers or by the addition of exogenous amylases. The interest of the study lies in the possibility of offering a naturally sweetened drink with no obligation to add sugar and which easily lends itself to pasteurization.

## 2. Material and Methods

### 2.1. Sample Origin

The experiments were carried out on tigernut tubers Ø > 1 cm bought in the market from the locality of Guili (Mokolo, Savannah Region of the Far North of Cameroon).

### 2.2. Sample Traitments

The tubers were subjected to soaking treatments in vitamin C solution (SVT), and part of the soaked tubers was subjected to sprouting (ST). Untreated tubers or Native Tubers (NT) were used as controls.

#### 2.2.1. Vitamin C Treatment 

This treatment consisted of soaking the tigernut tubers in the vitamin C solution (1 g/L) at 40 °C for 48 h, the time necessary for their maximum swelling.

#### 2.2.2. Tubers Sprouting

The tigernut tubers were sprouted according to the methods developed by Umerie et Enebeli [9] and Garcia Jiménez et al. [10]. For this purpose, the tubers soaked in the solution of vitamin C for 48 h at 40 °C were put for sprouting at 25 °C on jute bags protected from light for 6 days and sprayed with water two times a day (morning and evening). It should be noted that treatment with vitamin C is also a treatment for the destruction of molds (*Dematophora necatrix*), agents for the inhibiting germination of tubers [10]. After germination, the tubers were dried for 48 h at 40 °C in an oven and the rootlets removed manually. 

#### 2.2.3. Milk Extraction 

**Figure 1:**
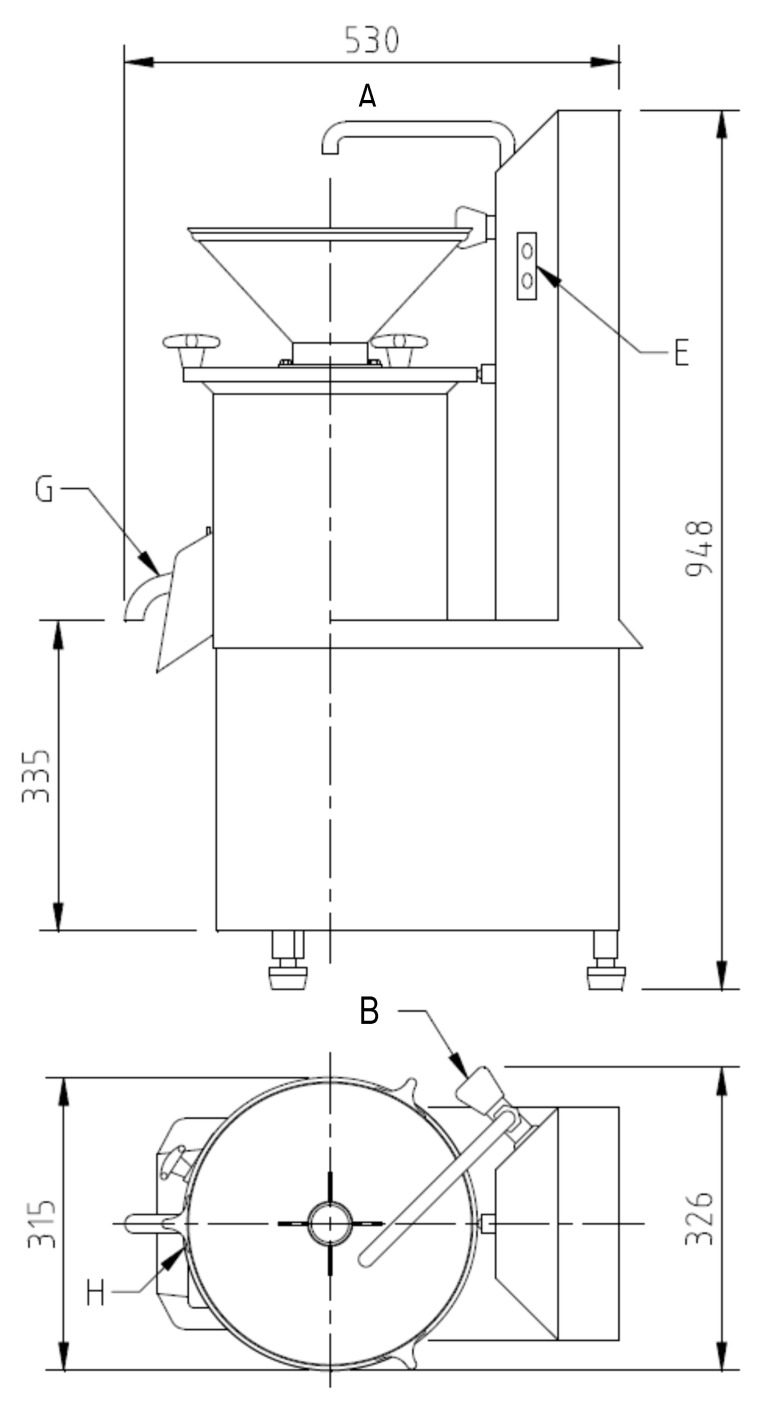
Diagram of the horchata machine (Moulin-Presse, H-MP 3) (**A**: front view; **B**: top view).

Different tigernut drinks ((NT), (SVT), and (ST)) were produced using a “horchata” machine (Moulin-Presse, H-MP 3, MEJISA MECTUFRY, la Mecanica Jijonenca Mectufry S.A.; Poligono de Segorb SN/03100 JIJONA, Alicante, Spain; Figure 1).

The hopper (C), where we pour the tigernut. Start button, (E). Tap, (F), to adjust the amount of water entering the machine. Milk discharge mouth or pipe, (G). Valve, (H), to empty the pressed pulp. Tank, (D), the interior of which contains a spinning wheel with grinding mallets; this rotating part allows grinding and maceration.

#### 2.2.4. In Situ Hydrolysis of Milk Starch

Starch in the three obtained beverage samples was subjected to hydrolysis using two sources of amylases: a commercial enzyme, Termamyl, which is an alpha amylase; and amylolytic extracts from the sprouted tigernut tubers.

- Termamyl Hydrolysis
  Termamyl is an enzyme of bacterial origin; its characteristics are listed in Table 1.
  To carry out this hydrolysis, 0.1 mL of Termamyl was applied to 100 mL of each sample contained in glass jars and kept under stirring conditions at the temperature of 80 °C in s water bath for 180 min.
- Amylolytic extracts hydrolysis
  - Amylolytic extracts preparation
    An amount of 25 g of sprouted tigernut tubers was powdered using the Dangoumeau-type mill (Dangoumill 300, Lonjumeau, France) in the presence of N_2_, and the powder obtained was sieved (500 μm). The powder obtained was transferred to a 250 mL flask and 150 mL of NaCl solution (5%) was added. The mixture was stirred for 15 min using the magnetic stirrer and at room temperature (25 °C). The mixture was filtered using Whatman No. 1 filter paper (9 cm), and the volume of the filtrate obtained was completed with NaCl (5%) at gauge line in the 250 mL flask. The enzyme extract was used after 2 h of rest.
  - Amylolytic extracts use
    Extracts from the sprouted tubers were used as a source of amylolytic enzymes for in situ hydrolysis of starch in milky drinks. The pH of the milky extracts was kept at 6.8 during the tests.
    To carry out the hydrolysis of starch contained in milky drinks, 0.1 mL of enzyme extract was applied to 100 mL of each sample after heating at 80 °C for 15 min and cooling at 45° C. The mixture was kept under stirring conditions in glass jars at 45 °C (a temperature which corresponds to the maximum activity of amylases of plant origin) [11] in the water bath for 180 min.
  - Hydrolysis kinetics
    In situ hydrolysis and kinetic analysis of starch in the different milk samples was carried out according to the methods of Goni et al. [12] and Bellmer et al. [13]. According to these methods, 1 mL of each sample was taken every 20 min for 180 min to determine the starch content in the reaction medium.

### 2.3. Physico-Chemical and Functional Analysis

#### 2.3.1. Amylolytic Activities of Tigernut Tubers

The amylolytic activities of the sprouted tiger tubers were determined by a spectrophotometric assay kit using amylase colorimetry (Megazyme International, County Wicklow, Ireland).

#### 2.3.2. Physico-Chemical Analysis

Examinations of the protein, crude fiber, and lipid contents were carried out by the AFNOR standard methods [14]. The total energy was obtained by the method of Edem et al. [15]. The dry matter and ash contents were determined by the Union Internationale de Chimie Pure et Appliquée (UICPA) methods [16]. The starch content was determined by the method of Mestres and Mestres [17]. The vitamin E and ascorbic acid levels were determined by the (NP)-HPLC method [18] and the Association of Official Analytical Chemists (AOAC) method [19], respectively. The total carbohydrates and reducing sugar were determined by AOAC [19] procedures.

#### 2.3.3. Rheological Profiles of Milks

Rheological measurements of the milky extracts were carried out on each sample using a dynamic rheometer (RHEOPLUS/32 V3.40 21004531-33056 Anton Paar, MCR 301 Physica, Virginia, USA). An amount of 15 g of each suspension of the milky extracts whose dry matter and starch contents varied from 17% to 19% and 3% to 12%, respectively, were subjected to a temperature ramp (from 50 to 90 °C at a speed of 2 °C/min), the initial temperature being fixed at 50 °C and gradually increasing in steps of 2 °C/min for a maximum of 90 °C. The temperature of the samples was maintained at this level for 5 min, then they were cooled to 50 °C in steps of 2 °C/min and maintained at this level for 3 min. The rheological profile data for the processed beverages was automatically saved by the computer connected to the rheometer and the rheological profile curves were also generated by the computer.

### 2.4. Statistical Analyses

The variation in the data collected and the statistical significance of the treatment effect were analyzed by an analysis of variance. Mean comparisons were made by Duncan’s test at the 5% probability level. The statistical data was analyzed using the STATGRAPHICS Centurion XVI.I (16.1.17) (2010) software, Virginia, USA and SigmaPlot 12.1 was used to plot the curves.

## 3. Results and Discussion

### 3.1. Influence of Sprouting on Physico-Chemical Characteristics and Functional Properties of Tigernut Tubers

Table 2 shows that sprouting results in a considerable reduction in the starch content, which is converted into reducing sugars because 66.66% increases in the concentration of these compounds were observed in the sprouted tubers compared to the native tubers. This observation was also reported by Umerie and Enibelie [9] and Ejoh et al. [20], who recorded a 67.34% increase in the concentration of reducing sugars after the sprouting of tigernut tubers for syrup preparation.

This process also increases the protein and ascorbic acid contents from 7.54 to 8.82 g/100 g DM and 250 to 275.39 mg/100 g DM, respectively. The increase in protein content could be related to the synthesis of enzymes, which are proteins [21]. As for the increase in the ascorbic acid content, Yudkin [22] and Laxmi et al. [23] pointed out that the germination of cereal and legume seeds was accompanied by an increase in the content of ascorbic acid, which in this case would justify the increase in the content of this parameter in the sprouted tubers. However, this treatment causes a slight reduction in lipid contents. This reduction could be explained by the fact that lipids are used to produce the energy necessary for the biochemical and physiological modifications intervening in the tuber during germination [24,25].

The interest in this process has been the stimulation or the synthesis of the amylolytic enzymes. The Table 2 shows that native tubers present only a slight amylolytic activity (3 U/mL). This activity increases during soaking. The highest activity is observed after tuber germination (60 U/mL) (Table 2). It was found that during this experiment, the amylolytic activity of the sprouted tubers increased from 3 to 60 U/mL, respectively, for NT and ST, which justifies the reduction in starch content and increase in the reducing sugar content at the end of germination. These results are similar to those of Traoré et al. [24], Chinma et al. [26], and Ojha et al. [27], who found that the native grains of sorghum, corn, and millet had no amylase activity, while the soaked and sprouted grains developed some amylase activity.

### 3.2. Extraction and Physicochemical Characterization of Milks: Influence of In Situ Hydrolysis of Tigernut Starch

#### 3.2.1. Extraction Yields of Tigernut Milk

**Figure 2:**
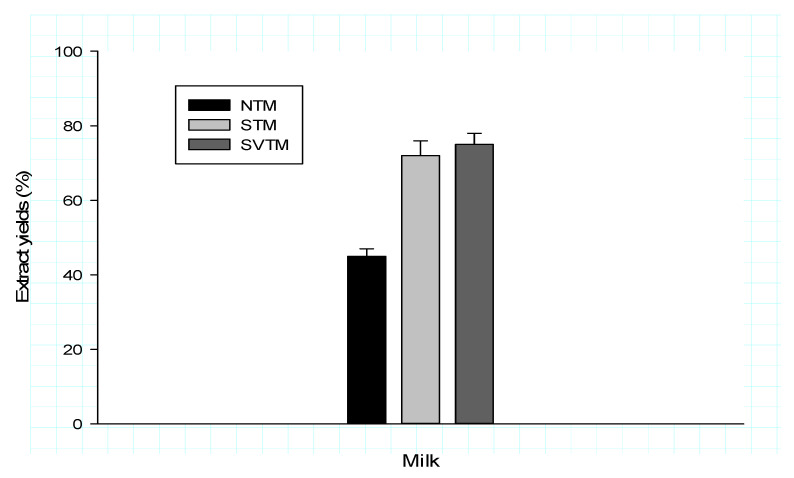
Extraction yields of milky suspensions for different processes applied to tigernut tubers. STM: Sprouted Tuber Milk; SVTM: Soaked in Vitamin C Tuber Milk; NTM: Native Tuber Milk (untreated).

Figure 2 shows the extraction yields of the tigernut milk obtained at the end of the process. The horchata machine of Spanish origin, used in the conditions of hardened and sprouted tubers, clearly improves the yield of milk extraction from tigernut tubers. The discontinuous process that had been used previously gave 13.79% yield extracts for the native tubers [2,19], whereas with the horchata machine (H-MP3, MEJISA MECTUFRY, Alicante, Spain), the yield is 45% for the same type of tubers, and an increase in yield of nearly 69.35% is observed. The sprouted tubers (ST) and soaked tubers (SVT) gave best yields of, respectively, 72% and 75%. This could be explained by the fact that these tubers are full of water and this water is found in milky drinks; it also facilitates the extraction of the drink [7]. In addition, soaked tubers (SVT) produce more milk than sprouted tubers (ST); this difference can be justified by the water content of the two types of tubers. Sprouted tubers have a 54.4% water content while soaked have about 57.3% [2].

#### 3.2.2. In Situ Hydrolysis Kinetics of Tigernut Milk Starch

**Figure 3:**
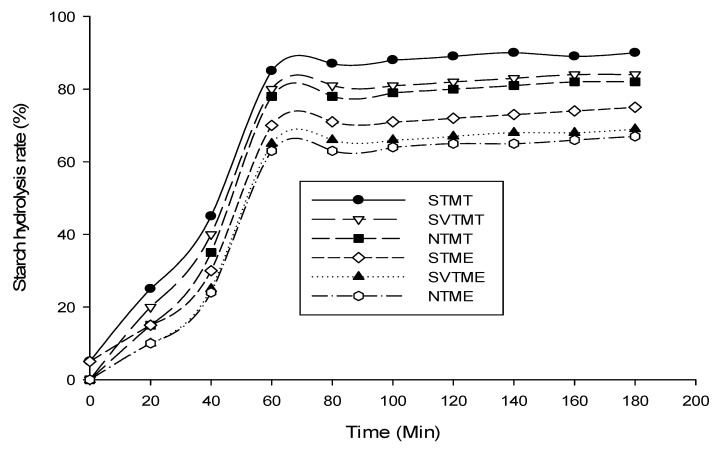
In situ starch hydrolysis rate of milky beverages of tigernut tubers. STMT: Sprouted Tuber Milk treated with Termamyl; SVTMT: Soaked in Vitamin C Tuber Milk treated with Termamyl; NTM T: Native Tuber Milk treated with Termamyl (untreated); STME: Sprouted Tuber Milk treated with amylolytic Extracts; SVTME: Soaked in Vitamin C Tuber Milk treated with amylolytic Extracts; NTME: Native Tuber Milk treated with amylolytic Extracts (untreated).

The hydrolysis of the starch in the milk of tigernut has been carried out using commercial enzymes (Termamyl), which include an alpha amylase and enzymes from the extracts of sprouted tubers. Figure 3 illustrates the starch hydrolysis rate of milk extracts, from native tubers (NTM), tubers soaked in vitamin C solution (SVTM), and sprouted tubers (STM), by these different enzymes. Termamyl has a stronger amylasic activity (85%) than the amylolytic extracts of sprouted tigernut tubers (75%). Termamyl has been used under its optimum conditions of activity, namely at the temperature of 80 °C, pH 6.8, which is that of tigernut milk, whereas the amylolytic extracts have been applied at the same pH (6.8) but at a temperature of 45 °C, which corresponds to the maximum activity temperature of plant-derived amylases according to the Handbook of Amylases [11] and Klang et al. [28]. Thus, this difference in the rate of hydrolysis can be attributed to the origins of the amylases (one bacterial and the other vegetable), the optimum temperature of activity, the pH conditions applied, the concentration, and the purity of the various enzymatic extracts.

The time required to achieve maximum starch hydrolysis is approximately 60 min, regardless of the source of amylase (Figure 3). This observation was previously reported by Jaisut et al. [29] and Klang et al. [28], who worked under the same conditions but on rice and sorghum starch respectively. Sprouting induces a decrease in the starch content and significantly influences the rate of starch hydrolysis by enzymes.

### 3.3. Chemical Composition of Milks

The different milky drinks obtained with/without the treatment of tigernut tubers as well as drinks having undergone the in situ hydrolysis of starch were analyzed to determine their chemical composition. Table 3 summarizes the results obtained. Drinks from sprouted tubers have higher protein and ascorbic acid levels of 4.22% and 54.93 mg/100 mL versus 2.47% and 23.07 mg/100 mL, respectively, for unsprouted tubers. However, their starch and lipid contents are lower than those of native tubers, being 4% and 6%, respectively, compared with 6% and 13%.

The milky extracts from tubers soaked in vitamin C solution (1 g/L) at 40 °C have a low fiber content (0.95% for SVTM against 2.03% for NTM), which can be explained by the fact that this treatment leads to the removal of tubercle tuberous, the film that covers the tuber, which has a cellulosic nature, meaning that its loss leads to a decrease in the fibers of the raw material. On the other hand, there is an increase in the ascorbic acid content of the order 75% relative to the milky extract of the untreated or native tuber, which is due to the phenomena of diffusion of this compound in the tuber during soaking in the vitamin C solution.

The different treatments applied to yellow nutsedge tubers (soaking in the ascorbic acid solution and germination) have an impact on the nutritional quality of beverages obtained from these tubers—in particular, the increase in the ascorbic acid and protein content, respectively, for soaking and germination, and a reduction in fiber in the case of soaking in ascorbic acid solutions, resulting in the peelings of yellow nutsedge tubers. These treatments also have effects on the sweetness of the various milky extracts, and this sweetness is reinforced by the hydrolysis of the starch contained in the beverages, as shown in Figure 5.

### 3.4. Sweetness of Milks

**Figure 4:**
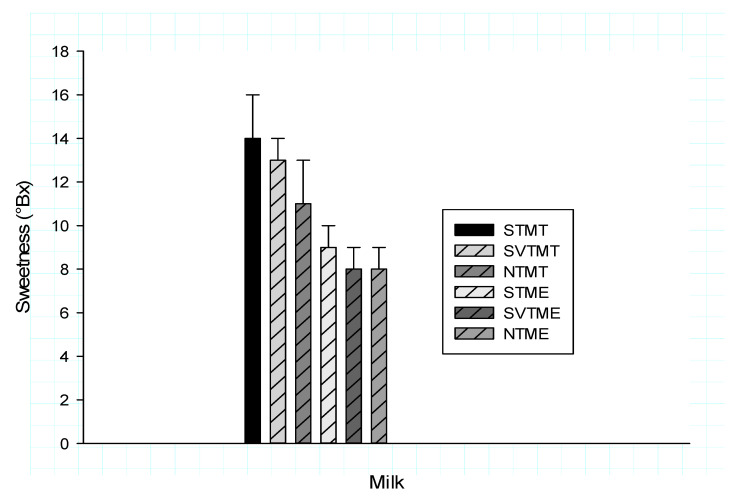
Tigernut milk sweetness during the hydrolysis of starch by amylolytic extracts and Termamyl. STMT: Sprouted Tuber Milk treated with Termamyl; SVTMT: Soaked in Vitamin C Tuber Milk treated with Termamyl; NTM T: Native Tuber Milk treated with Termamyl (untreated); STME: Sprouted Tuber Milk treated with amylolytic Extracts; SVTME: Soaked in Vitamin C Tuber Milk treated with amylolytic Extracts; NTME: Native Tuber Milk treated with amylolytic extracts (untreated).

Figure 4 illustrates the evolution of the sweetness of milky drinks from the yellow tigernut tubers following the hydrolysis of starch by Termamyl and amylase extracts. It appears that the sweetness of the drinks varies from 8 to 14°Bx as a function of the types of hydrolysis of the *C. esculentus* starch. The sprouted tubers have a higher sweetness than the non-sprouted tubers (TN) after hydrolysis, these results corroborate those of Otutu et al. [30] and Megat et al. [31]. Hydrolysis with Termamyl induces more free sugars. This process therefore significantly increases the sweetness of beverages compared to the hydrolysis of the amylase extracts of sprouted tubers.

At the end of these hydrolyses, Figure 4 shows three groups of milky drinks according to their degree of sweetness. The first group consists of beverages extracted from sprouted tubers whose starch has been hydrolyzed using Termamyl, this group has the highest degree of sweetness (14°Bx). The second group consists of beverages from sprouted tubers whose starch has been hydrolyzed using amylase extracts and native tubers whose starch has been hydrolyzed by Termamyl, which are moderately sweet (11°Bx). Other drinks have a degree of sweetness below 10°Bx.

As a result of these treatments, what can be the influences on the rheological profiles of these drinks?

### 3.5. Rheological Profile of Different Milks

The viscosity profile developed by the milky drinks of tigernut tubers as a function of time and temperature is shown in Figure 4. It represents a typical viscoamylographic characteristic profile of starch.

The rate of solubilization and the swelling phases as well as the temperature of the viscosity peak of tigernut starch are still poorly known. However, the work of Abdel-Akher and Michalinos [32] shows that tigernut starch viscosity is estimated at 2400 mPa.s, which is low compared to that of potato starch (7300 mPa.s) and maize (8400 mPa.s).

Under the hydrothermal effects of the treatment, the starch granules of the tigernut tubers begin to hydrate and swell after 200 s, inducing an increase in the viscosity of the suspension. Indeed, when the starch grains are heated to a sufficient temperature in the presence of an excess of water, the water penetrates the grains and causes their swelling (it is gelatinization). When the heating is prolonged, amorphous amylose is solubilized in the medium. The small amylose molecules are more easily released, while temperatures up to 90 °C are required to have a complete dispersion, including that of amyloses participating in the crystallization of amylopectin. The temperature at which the dispersion of the amyloses begins, which corresponds to the starch temperature of the *C. esculentus* tubers, is obtained at 77.8 °C.

During these phenomena of hydration and swelling of the granules, amorphous amylose is solubilized in the medium constituted by the tigernut tubers milk. The milk obtained is mainly composed of starch; these carbohydrates swell and constitute the dispersed phase and, in some cases, solubilized macromolecules (mainly amylose), which thicken the continuous phase. The rheological properties of the milk depend on the relative importance of these two phases and the swelling volume of the granules.

The viscosity peak is rapidly reached at about 314 mPa.s at 90.4 °C for native tuber extracts (NTM) and tubers treated with vitamin C (SVTM). This means that the milk contains a large number of highly swollen starch granules. When maintained at 90 °C, a viscosity break of 312 mPa.s appears at 600 s for NTM and SVTM and 118 mPa.s for the extracts of sprouted tubers (STM); meanwhile, the hydrolyzed beverages show no change after reaching the viscosity peak at 28 mPa.s.

When cooling to 50 °C, a significant increase in viscosity occurs in NTM, SVTM, and STM (336 mPa.s and 122 mPa.s). The final viscosities at 50 °C after 5 min are at 622, 207, and 27 mPa.s, respectively, for milk from native and soaked tubers (NTM, SVTM), milk from sprouted tubers (STM), and beverages whose starch has been subjected to enzymatic hydrolysis.

In fact, after heating the milky extracts will become, on cooling, more viscous and opaque; if the proportion of amylose is sufficient, it will gel. The firmness of the gel will be greater in the case of drinks obtained from the native and hardened tubers, which contain more amylose than the other extracts, hence the strong demotion of these drinks. There is no difference between the milk extracts from native tubers (NTM) and tubers treated with vitamin C (SVTM) for the hydrothermal characteristics of their starches.

The milky beverages whose starch has been subjected to hydrolysis do not show an increase in viscosity, and all have the same profile irrespective of the type of hydrolysis. These rheological profiles are different from those presented by Abdel-Akher and Michalinos [32], in the sense that these profiles testify to the presence of amylose in the starch structure (strong increase of the viscosity at cooling), whereas these authors had found that this entity would exist in the *C. esculentus* tubers starch in trace form (less than 1%).

The gelatinization temperature, which is defined as the temperature at which 50% of the grains lose their birefringence in polarized light, is 76 °C for starch of *C. esculentus* L., and that of early starch agglutination is 69 °C according to the work of Umerie and Uka [9]. These results are in line with those obtained in the context of this work, because the start of gelatinization temperature is 81.7 °C. The gelatinization temperature is a function of the size of the starch granules. The smaller the granules, the higher their gelatinization temperature [33]. It is for these reasons that the starch agglutination temperature of tigernut tubers milk is up to 90 °C. Hydration of *C. esculentus* starch granules at low temperatures below 60 °C is a reversible phenomenon that does not alter the physical properties of this starch. Hence the same gelatinization and starching temperature values for native and soaked tubers.

**Figure 5:**
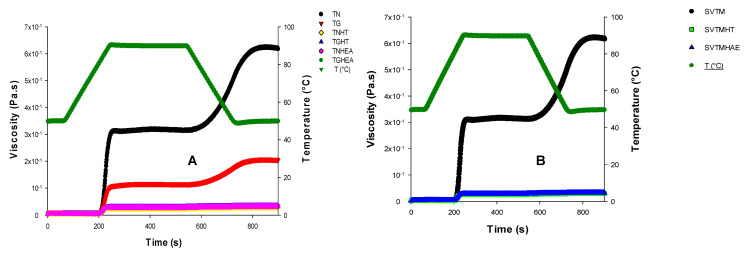
Rheological profile curves of milk extracts from native tubers (**A**) and tubers treated with vitamin C (**B**). NTM: Native Tubers Milk; STM: Sprouted Tubers Milk; STMHAE: Sprouted Tubers Milk treated with amylylolitic Extracts; NTMHT: Native Tubers Milk treated with Termamyl; STMHT: Sprouted Tubers Milk treated with Termamyl; NTMHAE: Native Tubers Milk treated with amylolytic Extracts; SVTM: Soaked Vitamin C Tubers Milk; SVTMHT: Soaked Vitamin C Tubers Milk treated with Termamyl and SVTMHAE: Soaked Vitamin C Tubers Milk treated with amylolytic extracts.

The results of Figure 3 and Figure 5 show us that tigernut tubers milk containing starch can be subjected to the hydrolysis treatment of this polysaccharide and these drinks can be pasteurized without major gelatinization and retrogradation phenomena. This is an important innovation in the production process of horchata.

## 4. Conclusions 

Under this study, the aim of which was to improve the yield and the practice extraction of tigernut milk through the production of this drink from sprouted tubers or by the addition of exogenous amylases, the following conclusions were drawn:

- The soaking and hydrolysis of the starch improves the milk extraction yield to almost 70% compared to untreated tubers.
- The different types of hydrolysis lead to an increase in the sweetness of the milky extracts.
- Amylolytic hydrolysis significantly reduces the starch content in the milk extracts and makes them suitable for pasteurization without caking tigernut milk, as shown by rheological profiles.

However, it is advisable to study the influence of pasteurization on the shelf life and physical appearance of tigernut milk over time. The influence of pasteurization on the nutritional and organoleptic qualities of the milky extracts after this treatment, as well as the quality of the packaging of treated tigernut milks, remain a scientific concern that will need to be addressed.

## Figures and Tables

**Table 1:** Characteristics of the exogenous enzyme used for starch hydrolysis.

Enzyme	Class	Origin	Optimal pH Activity	Optimal Temperature (°C)	Enzymatic Activity
Termamyl 120 L	α-amylase	*Bacillus licheniformis*	5.8–7	80–85	120 KNU g^−1^ *

* kg Novo α-amylase Unit.

**Table 2:** Chemical composition (g/100 gDM) of soaked (SVT), sprouted (ST), and native tubers (NT).

Characteristics (g/100 g DM)	Sprouted Tubers	Tubers Treated with Vitamin C	Native Tubers (Control Sample)
Water content (%)	54.38 ± 0.54 ^b^	57.34 ± 0.23 ^c^	7.38 ± 0.14 ^a^
Protein	8.82 ± 1.31 ^a^	7.42 ± 0.42 ^b^	7.62 ± 0.11 ^b^
Total carbohydrates	48.93 ± 1.18 ^a^	47.52 ± 1.84 ^a^	49.92 ± 0.12 ^b^
Reducing sugars	33.36 ± 0.35 ^a^	23.74 ± 1.74 ^b^	20.12 ± 1.11 ^c^
starch	16.63 ± 0.50 ^b^	25.13 ± 0.10 ^a^	26.14 ± 0.27 ^a^
Lipids	24.15± 0.02 ^b^	26.25 ± 0.53 ^a^	25.56 ± 0.41 ^a^
Fibers	15.72 ± 0.09 ^a^	12.03 ± 0.94 ^c^	15.56 ± 0.12 ^a^
Ashes	3.84 ± 0.18 ^a^	1.84 ± 0.07 ^b^	2.73 ± 0.31 ^a^
Vitamin C (mg/100 g)	275.39 ± 3.41 ^b^	328 ± 4.37 ^a^	252 ± 0.39 ^c^
Vitamin E (mg/100 g)	118.73 ± 0.55 ^a^	118.79 ± 3.26 ^a^	123 ± 0.18 ^b^
Caloric Value (kcal)	462	450	445
AA * (U/mL)	60 ± 3.72 ^a^	15 ± 0.58 ^b^	3 ± 1.69 ^c^

Results with the same letters exponent on the same line are not significantly different (probability threshold *p* ≤ 0.05) * AA: Amylolytic activity; U (unit of amylase activity): is defined as equivalent to the release of 1 μg of glucose per minute and per mL.

**Table 3:** Chemical composition of milk extracts from hydrolyzed and unhydrolyzed tigernut tubers, before and after sprouting.

Parameters (%)	STM	SVTM	STMT	STME	SVTMT	SVTME	NTM
Dry matter	19.22 ± 1.63 ^c^	18.52 ± 0.43 ^b^	18.78 ± 1.48 ^b^	18.82 ± 1.93 ^b^	18.62 ± 1.15 ^b^	18.56 ± 1.54 ^b^	17.41 ± 1.52 ^a^
Ashs	0.92 ± 0.03 ^b^	0.90 ± 0.09 ^b^	0.97 ± 0.07 ^b^	0.94 ± 0.07 ^b^	0.91 ± 0.08 ^b^	0.92 ± 0.07 ^b^	0.70 ± 0.03 ^a^
Proteins	4.22 ± 0.54 ^b^	2.45 ± 0.97 ^a^	3.89 ± 0.72 ^b^	3.75 ± 0.73 ^b^	2.78 ± 0.54 ^a^	2.68 ± 0.37 ^a^	2.47 ± 0.75 ^a^
Starch	7.57 ± 1.44 ^b^	12.98 ± 1.70 ^a^	3.85 ± 1.52 ^c^	4.77 ± 0.95 ^c^	3.96 ± 0.33 ^c^	4.85 ± 0.59 ^c^	12.87 ± 1.95 ^a^
Lipids	4.11 ± 0.68 ^c^	5.83 ± 0.71 ^b^	3.95 ± 0.22 ^c^	4.03 ± 0.06 ^c^	5.06 ± 0.66 ^b^	5.53 ± 0.74 ^b^	6.33 ± 0.31 ^a^
Vitamin C (mg/100 mL)	54.93 ± 1.85 ^c^	92.62 ± 2.22 ^a^	12.91 ± 3.87 ^f^	52.49 ± 1.19 ^c^	17.35 ± 1.09 ^e^	91.28± 2.37 ^b^	23.07 ± 1.85 ^d^
Vitamin E (mg/100 mL)	7.26 ± 1.66 ^a^	7.76 ± 2.65 ^a^	7.37 ± 1.74 ^a^	6.85 ± 0.54 ^a^	7.90 ± 1.74 ^a^	7.94 ± 1.82 ^a^	6.16 ± 1.98 ^b^
Fibers	1.97 ± 0.54 ^a^	0.95 ± 0.06 ^b^	1.99 ± 0.08 ^a^	1.93 ± 0.11 ^a^	0.97 ± 0.23 ^b^	0.97 ± 0.05 ^b^	2.03 ± 0.53 ^a^

Results with the same letters exponent on the same line are not significantly different (probability threshold *p* ≤ 0.05). STMT: Sprouted Tuber Milk treated with Termamyl; SVTMT: Soaked in Vitamin C Tuber Milk treated with Termamyl; NTM T: Native Tuber Milk treated with Termamyl (untreated); STME: Sprouted Tuber Milk treated with amylolytic Extracts; SVTME: Soaked in Vitamin C Tuber Milk treated with amylolytic Extracts; NTME: Native Tuber Milk treated with amylolytic Extracts (untreated).
